# Association of Obesity Indices with Diabetic Kidney Disease and Diabetic Retinopathy in Type 2 Diabetes: A Real-World Study

**DOI:** 10.1155/2023/3819830

**Published:** 2023-04-15

**Authors:** Shaomin Shi, Lihua Ni, Yuan Tian, Baifang Zhang, Jing Xiao, Wan Xu, Ling Gao, Xiaoyan Wu

**Affiliations:** ^1^Department of Nephrology, Zhongnan Hospital of Wuhan University, 169 Donghu Road, Wuhan, Hubei 430071, China; ^2^Xiangyang Central Hospital, Affiliated Hospital of Hubei University of Arts and Science, Xiangyang, Hubei 441000, China; ^3^Department of Biochemistry, Wuhan University TaiKang Medical School (School of Basic Medical Sciences), Wuhan, Hubei 430071, China; ^4^Department of General Practice, Zhongnan Hospital of Wuhan University, 169 Donghu Road, Wuhan, Hubei 430071, China

## Abstract

**Background:**

Diabetic microvascular complications mainly include diabetic kidney disease (DKD) and diabetic retinopathy (DR). Obesity was recognized as a risk factor for DKD, while the reported relationship between obesity and DR was inconsistent. Moreover, whether the associations can be attributed to C-peptide levels is unknown.

**Methods:**

Data from 1142 sequential inpatients with T2DM at Xiangyang Central Hospital between June 2019 and March 2022 were extracted retrospectively from the electronic medical record system. The associations between four obesity indices (body mass index (BMI), waist-hip circumference ratio (WHR), visceral fat tissue area (VFA), and subcutaneous fat tissue area (SFA)) and DKD and DR were evaluated. Whether the associations can be attributed to C-peptide levels was also explored.

**Results:**

Obesity was a risk factor for DKD after adjusting for sex, HbA1c, TG, TC, HDL, LDL, smoking history, education, duration of diabetes, and insulin use (obesity indices: BMI (OR 1.050: 95% CI: 1.008-1.094; *P* = 0.020); WHR (OR 10.97; 95% CI: 1.250-92.267; *P* = 0.031); VFA (OR 1.005; 95% CI: 1.001-1.008; *P* = 0.008)), but it became insignificant after further adjusting for fasting C-peptide. The associations between BMI, WHR, VFA, and DKD might be U-shaped. Obesity and FCP tended to protect against DR; however, they became insignificant after adjusting for multiple potential confounders. C2/C0 (the ratio of the postprandial serum C-peptide to fasting C-peptide) was a protective factor for both DKD (OR 0.894, 95% CI: 0.833-0.959, *P* < 0.05) and DR (OR 0.851, 95% CI: 0.787-0.919; *P* < 0.05).

**Conclusions:**

Obesity was a risk factor for DKD, and the effect may be attributable to C-peptide, which represents insulin resistance. The protective effect of obesity or C-peptide on DR was not independent and could be confounded by multiple factors. Higher C2/C0 was associated with both decreased DKD and DR.

## 1. Introduction

Obesity is considered to be one of the established risk factors for diabetes mellitus (DM) and the development of vascular complications [1, 2]. Diabetic microvascular complications, including diabetic kidney disease (DKD) and diabetic retinopathy (DR), are severely associated with reduced quality of life and increased mortality [3]. A close relationship between DKD and DR has been presented in epidemiologic studies [4] since they have similar structural and physiological changes [5, 6]. However, in the real world, the presentations of DR and DKD are not always consistent, suggesting differences in their pathogenesis [4, 7]. Obesity was reported to correlate positively with DKD, while the reported relationship between obesity and DR was inconsistent in previous studies [8–10]. Some studies indicated that obesity increased DR risk [11]; however, other recent studies suggested either no association [9] or a negative association [8, 12]. Recently, some evidence suggested that fat distribution rather than general obesity was associated with DKD or DR [13, 14], but these findings are still inconclusive [1].

Body mass index (BMI), as the most commonly used parameter to assess overweight or obesity, may not be a good marker for distinguishing general obesity and central obesity. WHR (waist-hip circumference ratio), as an index of body fat distribution, is commonly used to assess central obesity but is unable to distinguish between visceral and subcutaneous fat. Visceral fat tissue area (VFA) and subcutaneous fat tissue area (SFA) measurements can further distinguish the sources of central obesity [14]. Subcutaneous fat is stored under the skin, while visceral fat is mainly located in the abdominal cavity, where it can release its components (fatty acids and adipokines) into the venous system to affect systemic metabolism [15, 16]. Visceral adipose tissue plays a key role in the development of obesity-related diseases, while the role of subcutaneous adipose tissue is always less significant. Studies focused on the associations of combined obesity indices and DKD or DR are limited with inconclusive results, and comparisons between DKD and DR are even more scarce.

Furthermore, obesity might be involved in the pathogenesis of DKD or DR through insulin resistance, inflammation, endothelial dysfunction, fibrinolysis, and thrombosis [17]. C-peptide, which is cosecreted with insulin in an equimolar ratio, is a known indicator of pancreatic *β*-cell function or insulin resistance [18]. C-peptide was reported to be associated with diabetic microvascular complications [19]. A previous study reported that the association between obesity and DR may partially contribute to C-peptide levels [12]. However, to our knowledge, no study has reported the effect of obesity on DKD in association with C-peptide, and there are still many gaps in our understanding.

Thus, this study is aimed at assessing and comparing the associations of combined obesity indices with DKD and DR in patients with type 2 diabetes mellitus (T2DM) and exploring whether the associations can be attributable to C-peptide levels. Our study may provide evidence for clinical treatment and shed light on further study of the pathogenesis of DKD and DR.

## 2. Materials and Methods

### 2.1. Study Design and Population

This was a retrospective cross-sectional study in the real world. Data from 1208 sequential inpatients with T2DM from Xiangyang Central Hospital between June 2019 and March 2022 were extracted from the electronic medical record system. Inclusion criteria are adult DM and hospitalized patients who took fundus photographs and had waist circumference (WC) and hip circumference (HC) measured. Excluded criteria are DM who were not classified as T2DM (*n* = 17), severe systemic diseases (such as eGFR≤15 ml/min/1.73 m^2^, severe heart failure, severe liver disease, or malignant tumor) or acute complications of DM (such as diabetic ketoacidosis, hyperglycemic hyperosmolar coma, lactic acidosis, or hypoglycemia coma) (*n* = 7), glaucoma (*n* = 1), and patients without accessible data (*n* = 41). Finally, 1142 patients were included.

The study protocol was approved by the Ethics Committee of Xiangyang Central Hospital, an affiliated hospital of Hubei University of Arts and Science. The study was performed in accordance with the guidelines of the Declaration of Helsinki. Private personal information was removed during the process of analysis and publication. Informed consent exemptions were approved by the ethics committees. Ethics batch number: XYSZXYY-LLDD-PJ-2022-081. Clinical trial registration number: ChiCTR2200060132.

### 2.2. Demographic Information, Medical History, and Biometric Parameter Collection

Demographic information and medical history were extracted, including age, sex, hypertension history, smoking history, education level (≥high school or not), duration of DM, CVD history (defined as a history of cardiovascular and cerebrovascular disease), ever insulin use, systolic and diastolic blood pressure (SBP, DBP), weight, height, WC, HC, visceral fat area (VFA), and subcutaneous fat area (SFA). Biometric information extracted included fasting blood glucose (FBG), fasting C-peptide (FCP), 2 h postprandial C-peptide (PCP), glycated hemoglobin (HbA1c), serum lipids (triglyceride (TG), total cholesterol (TC), high-density lipoprotein (HDL), low density lipoprotein (LDL)), serum creatinine, and urinary albumin to creatine ratio (UACR).

The weight and height of the participants were measured using a weight scale, with their shoes and heavy objects removed. WC and HC were measured using medical tape. WC was measured along the minimum perimeter between the costal and anterior superior iliac spine at the end of expiration. HC was taken at the maximum circumference of the hip. VFA and SFA were measured by the bioelectrical impedance technique (Omron HDS-2000). Blood pressure was measured using a mercury sphygmomanometer after sitting for at least 5 minutes. Blood samples were collected after an overnight fast (≥8 h) and at 120 min following a diabetic diet. Morning spot urine samples were drawn. All samples were cold chained and transported to a central laboratory for testing within 2-4 h.

The levels of HbA1c were measured using high-performance liquid chromatography (MQ-2000PT, Medconn, China). FBG, serum creatine, TG, TC, HDL, LDL, and UACR were measured by standard methods, with a Beckman Coulter AU 680 (Brea, USA). The levels of C-peptide were measured by radioimmunoassay (Linco Research, St Charles, MO, USA).

The C2/C0 ratio was defined as PCP divided by FCP. HOMA-IR was calculated as follows: HOMA − IR = 1.5 + FBG (mmol/L) ^∗^FCP (pmol/L)/2800 [20]. BMI was calculated as weight divided by height squared. WHR was calculated as WC divided by HC.

### 2.3. Definition of DKD and DR

The estimated glomerular filtration rate (eGFR) was evaluated according to the Chinese Chronic Kidney Disease Epidemiology Collaboration (CKD-EPI) formula as follows: eGFR (ml/min/1.^73m2^) = 175 × (serum creatinine mg/dL)^−1.154^ × (age)^−0.203^ (×0.742 if female) [21]. DKD was defined as a history of T2DM and eGFR less than 60 ml/min/1.73 m^2^ or albuminuria more than 30 mg/g, after ruling out other possible causes of kidney injury (such as acute kidney injury and primary glomerulopathy) [22].

Color fundus photographs were taken using a retinal fundus camera (Canon, CR-2, Japan) at 45° of both eyes without mydriatic agents for each patient. Fundus photographs were read by experienced ophthalmologists. DR was diagnosed and graded according to the consensus of the global diabetic retinopathy project group (DR presented as microaneurysms, hemorrhages, venous beading, prominent intraretinal microvascular abnormalities, neovascularization, or vitreous/preretinal hemorrhages) [23].

### 2.4. Statistical Analysis

Stata Version 16 (Stata Corporation, College Station, TX, USA) was used for all the analyses. Normally distributed continuous variables were presented as the mean ± standard deviation, while nonnormally distributed variables were presented as the median with an interquartile range (25%, 75%). The t test was used to analyze the differences when they were normally distributed; if not, the Mann–Whitney *U* test was used. Categorical variables were reported as counts with proportions (%), and the chi-square test was used for different comparisons.

Logistic regression models were used to assess the associations between the parameters and DR and DKD. The confounding variables with collinearity, tested by a calculated variance inflation factor (VIF) ≥10, were removed from the modified model. Thus, modified model 1 adjusted for sex, HbA1c, TG, TC, HDL, LDL, smoking history, education, duration of diabetes, and insulin use. Modified model 2 further adjusted for FCP. In addition, associations were also assessed by quartiles. Ordinal logistic regression models were used to assess the overall trend of the categorical variables with the presence of DKD and DR. We also used the restricted cubic spline function fitted for logistic regression models with 4 knots at the 5-25th, 50th, 75th, and 95th percentiles of obesity indices. Few missing data were not included in the analysis (education level missed 7.9%, HbA1c missed 6.1%, DR diagnosis missed 3.2%, lipids missed 3.3%, and C-peptide, subadipose, inneradipose, FBG, smoking history, ever insulin use, weight, and height missed <2%, without any other data missed). A *P* value less than 0.05 (two sided) indicated significance.

Multiple sensitivity analyses were also performed. To further analyze the associations of obesity with DKD/DR, missing values (HbA1c, serum lipids, FCP, VFA, SFA, FBG, weight, and height) were imputed and replaced by the mean. Moreover, FCP was replaced by HOMA-IR, and gender-stratified analyses were also performed.

## 3. Results

### 3.1. Basic Characteristics

A total of 1142 cases were enrolled, and 69.8% of patients with type 2 diabetes were overweight or obese. In general, the overall median age of the participants was 56 years old. The prevalence of DKD was 32.2% (5.8% of them had eGFR<60 ml/min.1.73 m^2^, and 7.4% of them had UACR>300 mg/g), while the prevalence of DR was 30.7%. The prevalence of DR in patients with DKD and that of DKD in patients with DR both increased to 43.2% and 44.1%, respectively.

Compared with patients without DKD or DR, those with DKD or DR both had older age, longer duration of diabetes, higher SBP, more frequent hypertension history, a higher proportion of ever insulin use, higher UACR, and lower eGFR. Additionally, they both had higher HbA1c, with DR patients having a more significant difference. Inconsistently, patients with DKD had higher BMI, higher TG, and more frequent CVD history, while patients with DR had lower LDL and lower education level and tended to have lower BMI, but the difference was not significant. Regarding other obesity indices, those with DKD had higher levels (WC, HC, WHR, and VFA), while those with DR presented lower levels (WC, HC, VFA, and SFA). DKD patients had higher FCP and HOMA_IR, while DR patients had lower values. However, C2/C0 was lower in patients with either DKD or DR (all *P* < 0.05) (as shown in Table 1).

### 3.2. Association between Obesity Indices and DKD

In the base unadjusted model, logistic models showed that obesity was a risk factor for DKD (obesity indices: BMI (odds ratio OR, 1.052; 95% CI 1.058-1.090; *P* = 0.005); WHR (OR 25.64; 95% CI 4.148-158.473; *P* < 0.05); VFA (OR 1.006; 95% CI 1.003-1.009; *P* < 0.05); SFA (OR 1.002; 95% CI 0.999-1.004; *P* = 0.078)), with WHR having the most significant influence, and the influence of SFA was not significant. In adjusted model 1, after adjusting for sex, HbA1c, TG, TC, HDL, LDL, smoking history, education level, duration of diabetes, and ever insulin use, the significant associations were attenuated and reserved. However, in model 2, after further adjusting for FCP, the associations were no longer significant (as shown in Table 2). Additionally, the associations disappeared when stratified by FCP quartiles (as shown in Supplementary Table 1).

**Figure 1:**
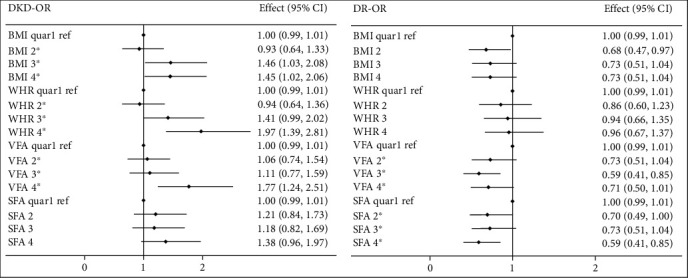
The associations between obesity indices and DKD/DR by quartiles. ^∗^*P* for trend <0.05. Abbreviations: DKD indicates diabetic kidney disease; DR: diabetic retinopathy; BMI: body mass index; WHR: waist to hip circumference ratio; VFA: visceral fat area; SFA: subcutaneous fat area; Quar: quartile; Ref: reference; CI: confidence interval.

In the quartile regression analyses, in base unadjusted models, increasing obesity indices increased the prevalence of DKD: BMI (OR 1.054, 95% CI 1.014-1.096; *P* for trend = 0.008), WHR (OR 72.609, 95% CI 10.028-5525.750; *P* for trend < 0.001), and VFA (OR 1.006, 95% CI 1.003-1.009, *P* for trend = 0.001). However, the influence of SFA was not significant (OR 1.002, 95% CI 0.999-1.004, *P* for trend = 0.097). Moreover, compared with other quartiles, BMI quartile 2 (23.4-25.6 kg/m2) and WHR quartile 2 (0.897-0.944) had the lowest risk for both DKD and DR but without statistical significance (details of associations by quartiles shown in Figure 1).

### 3.3. Association between Obesity Indices and DR

In base unadjusted models, logistic models showed that obesity was not associated with DR. Although VFA (OR 0.996, 95% CI 0.993-0.999; *P* = 0.023) and SFA (OR 0.997, 95% CI 0.996-1.000; *P* = 0.007) tended to be protective factors for DR, the associations became insignificant after adjusting for potential confounders (sex, HbA1c, TG, TC, HDL, LDL, smoking history, education, duration of diabetes, and insulin use or FCP) (as shown in Table 2).

In the quartile regression analyses, VFA (OR 0.996, 95% CI 0.993-0.999; *P* for trend = 0.045) and SFA (OR 0.997, 95% CI 0.995-0.999; *P* = 0.008) tended to be protective factors for DR (as shown in Figure 1), but the associations became insignificant after adjusting for potential confounders (*P* > 0.05).

## 4. Restricted Cubic Spline Figures of Obesity Indices to DKD/DR Using Logistic Regressions

**Figure 2:**
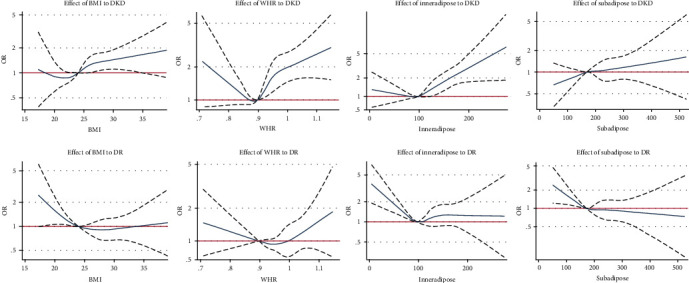
Restricted cubic spline figures of obesity indices to DKD and DR using logistic regressions.

Restricted cubic spline figures indicated that with increasing SFA, the prevalence of DR tended to decrease, and the prevalence of DKD tended to increase. However, the associations between the other three obesity indices (BMI, WHR, and VFA) and DKD or DR were all U-shaped. The risk of DKD or DR decreased initially and reached the lowest, when BMI was 20.80-22.01 kg/m^2^ for DKD and 26.27-29.01 kg/m^2^ for DR, respectively, when WHR was 0.879-0.889 and 0.903-0.917, respectively, and when VFA was 88.4-91.8 cm^2^ and 100.5-102.4 cm^2^, respectively (as shown in Figure 2).

### 4.1. Association between C-Peptide and DKD/DR

**Figure 3:**
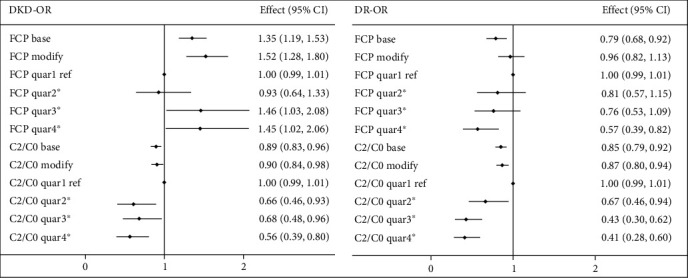
The associations between C-peptide levels and DKD/DR. ^∗^*P* for trend <0.05. Abbreviations: DKD indicates diabetic kidney disease; DR: diabetic retinopathy; FCP: fasting C-peptide; C2/C0: the ratio of FCP to PCP; Quar: quartile; Ref: reference; CI: confidence interval. Modified model adjusted for age, gender, SBP, HbA1c, TG, TC, HDL, LDL, smoking history, education, duration of diabetes, ever insulin use, and BMI.

Logistic models showed that increasing FCP was a risk factor for DKD (OR 1.350, 95% CI 1.189-1.532; *P* < 0.05), and increasing C2/C0 was a protective factor for both DKD (OR 0.894, 95% CI 0.833-0.959; *P* < 0.05) and DR (OR 0.851, 95% CI 0.787-0.919; *P* < 0.05), even after adjusting for all potential confounders (age, sex, SBP, HbA1c, TC, TG, LDL, HDL, smoking history, education, duration of diabetes, ever insulin use, and BMI). However, although increasing FCP was a protective risk factor for DR (OR 0.790, 95% CI 0.681-0.916; *P* < 0.05), the protective effect became insignificant after adjusting for potential confounders (OR 0.965, 95% CI 0.823-1.132; *P* = 0.662) (as shown in Figure 3).

When analyzed by quartiles, in base unadjusted models, increasing FCP increased the risk of DKD (OR 1.35, 95% CI 1.16-1.56; *P* for trend < 0.05) and decreased the risk of DR (OR 0.79, 95% CI 0.67-0.92; *P* for trend = 0.003). Increasing C2/C0 protected against both DKD (OR 0.90; 95% CI 0.84-0.97; *P* for trend = 0.006) and DR (OR 0.83, 95% CI 0.77-0.90; *P* for trend < 0.05) (as shown in Figure 3).

### 4.2. Supplementary Analysis

Subgroup analysis showed that obesity indices increased the risk of DKD (BMI, WHR, and VFA) and decreased the risk of DR (BMI and VFA), only within a certain range (as shown in Supplementary Table 2).

We replaced FCP with the index of insulin resistance (HOMA-IR), and the results of the associations between obesity indices and DKD were the same (as shown in Supplementary Table 3). Increasing HOMA-IR was a risk factor for DKD after adjusting for all potential confounders (OR 1.362 (1.179, 1.574), *P* < 0.001), and it was not significantly associated with DR. Additionally, no gender difference was found in the present study (as shown in Supplementary Table 4). When the few missing values were replaced by the mean (HbA1c, lipids, FCP, VFA, SFA, FBG, weight, and height), the results remained almost consistent with those before (as shown in Supplementary Table 5). Moreover, C2/C0 was replaced by C2_C0 (calculated as postprandial C − peptide minus fasting C − peptide). The protective effect for DR was consistent with before, but that for DKD became insignificant (Supplementary Table 6).

## 5. Discussion

The present study included a large hospital-based population. We evaluated and compared the associations of four different obesity indices with DKD/DR and the concentration of C-peptide. The following are the main findings: first, the prevalence of overweight or obesity in T2DM is as high as 69.8%. Second, obesity was a risk factor for DKD and might be attributable to C-peptide, which represented insulin resistance. Third, the protective effect of obesity (VFA and SFA) or FCP on DR was not independent, which might be confounded by multiple factors. Fourth, the associations between BMI, WHR, VFA, and DKD or DR might be U shaped. Fifth, C2/C0 was a protective factor for both DKD and DR.

### 5.1. Effects of Obesity on DKD

Our study showed that obesity and FCP might be risk factors for DKD, which was consistent with previous studies [9, 13, 20, 24, 25]. Obesity might promote DKD by promoting insulin resistance, the inflammatory response, endothelial dysfunction, arteriosclerosis, fibrosis, thrombosis, and so on. The relationship between insulin resistance and DKD is complex. Insulin resistance commonly promotes DKD in the early stage and is almost universal in end-stage renal disease (ESRD) through multiple mechanisms [26]. Moreover, obesity was accompanied by increased ectopic fat deposition in the pararenal vascular or renal sinuses, which might further promote DKD [17, 27]. FCP has long been recognized to represent insulin resistance and can be used to calculate the insulin resistance index [20]. C-peptide itself can also affect various steps in the process of atherosclerosis [28]. In addition to greater FCP levels and insulin resistance, obese patients with T2DM always have elevated BP and worse lipid profiles simultaneously, which are all potential contributors to DKD [17, 27]. BMI may not be accurate enough for measuring obesity since it induces both muscle and bone mass [29]. WHR may be a more sensitive marker for obesity measurement. Consistently, in our study, WHR had a much more significant influence than BMI (OR 25.64, 95% CI 4.146-158.473 vs. 1.052 95% CI 1.058-1.090), which also indicated that abdominal obesity had a more detrimental effect on DKD than generalized obesity. Moreover, although obesity was a risk factor for DKD, the association became insignificant after adjusting for FCP or stratifying by FCP quartiles, which hinted that the effect may be mediated by FCP.

### 5.2. Effects of Obesity on DR

In previous studies, the relationship between obesity and DR was reported inconsistently (positive, negative, or none) [10, 30, 31]. Hwang et al. reported that total body fat assessed by dual-energy X-ray absorptiometry was associated with DR [32]. Dossarps et al.'s study included 179 patients with T2DM and showed that visceral fat and subcutaneous fat measured by MRI were not associated with DR [33]. The discrepancy might be explained by the differences in study methodology, study design, sample size, and study participants. It is worth noting that studies showing a higher BMI-increased risk for DR always included white diabetic patients, and most Asian studies showed no association or an inverse association between obesity and DR.

Increasing attention has been given to the role of C-peptide itself. It was reported that C-peptide was not only a byproduct of insulin secretion but also a bioactive peptide with both endocrine functions [34], such as the activation of endothelial nitric oxide synthase and Na+/K+/ATPase, as well as a variety of transcription factors [35, 36]. Therefore, some studies have reported that C-peptide is a protective factor against diabetic complications, and exogenous C-peptide supplementation can prevent or alleviate vascular and neurological complications [37, 38].

However, in the present study, although obesity (VFA and SFA) and FCP presented protective effects for DR, they disappeared after adjusting for multiple confounding factors (including HbA1c, DM duration, FCP, and CVD history), which indicated that obesity or FCP was not an independent protective factor for DR. Obesity or increased FCP indicated shorter duration, better islet function, and glycemic control and was therefore associated with fewer complications [39]. Thus, the protective effect of obesity or FCP on DR might be confounded by better *β* cell function and blood glucose control, shorter duration, and so on. Consistently, Klein et al. found that the protective effect of FCP on DR became insignificant after controlling HbA1c [40]. Wu et al.'s study showed that the protective effect of visceral adiposity on incident DR became insignificant after adjusting for confounding factors [13].

### 5.3. Comparison of the Paradoxical Correlations of Obesity with DKD and DR

As we know, DKD and DR are both microvascular complications of T2DM [41]. A close relationship between them has been reported in epidemiologic studies [4] since they share similar structural and physiological changes [6]. However, similar to some previous studies, our study showed that obesity and higher FCP were associated with an increased risk of DKD but tended to decrease the risk of DR [20, 24, 25]. The discrepant role of obesity and FCP in the development of diabetic vascular complications is worth mentioning.

On the one hand, the effect of obesity on diabetic complications may be two-sided, with both positive and negative effects, and FCP was correlated with both insulin resistance and insulin secretion defects, as discussed before. The final effect on a certain organ might depend on the overall interactions between them. The kidney has many high-affinity insulin receptors, which are widely expressed in renal tubular cells and podocytes. Insulin signaling plays an important role in podocyte activity and renal tubular cell function. When insulin is resistant, glomerular insulin signaling is impaired, and the mitogen-activated protein kinase pathway stimulates vasoconstriction. Insulin resistance reduces adiponectin secretion and clearly increases leptin; the former promotes epithelial cell dysfunction and proteinuria, and the latter promotes renal fibrosis through TGF-*β* [42]. Thus, insulin resistance may be the vital pathological component in DKD, which is always accompanied by higher FCP or obesity. However, for retinal effects, the beneficial effects of obesity may counteract or attenuate the detrimental effect, which could be a protective factor. Obesity always indicated better islet function and glycemic control. In fact, insulin deficiency and glycemic control might play a crucial role in the development of DR. Evidence has shown that type 1 diabetes is more frequently complicated with DR than T2DM (approximately 99% vs. 60% 20 years after the onset of diabetes), which may contribute to better glycemic control in T2DM [43]. Another piece of evidence is that it was reported that hyperglycemia-induced DR can be reversed by strict glycemic control [44]. Moreover, Ahlqvist et al. reported that diabetic patients in the severe insulin deficiency group had an increased risk of DR and neuropathy, whereas those with severe insulin resistance had the highest risk for DKD, and the severe insulin resistance subtype developed DKD independent of metabolic control and HbA1c [26].

On the other hand, in addition to consistency, differences also existed between DKD and DR, which might contribute to the paradoxical correlations of obesity with DKD/DR. For example, neuropathy was involved in the development of DR instead of DKD. As a part of the peripheral nervous system, the optic nerve is sensitive to hypoxia, ischemia, and metabolic disorders and is easily impaired, which might appear early and continue throughout [45]. Additionally, both DKD and DR have a genetic predisposition, but the susceptibility genes and genetic background are different [46]. In addition, cytokines that have been reported to be involved in the pathogenesis of DKD and DR are not completely consistent [47].

In addition, restricted cubic spline figures in the present study showed that a U-shaped association of obesity with DKD and DR may exist, consistent with Lu et al.'s study [12]. Obesity was a risk factor for DKD and a protective factor for DR when above or below a certain range, but when the obesity indices were too low or too high, the effect was opposite or insignificant.

### 5.4. The Influence of Altered Serum C-Peptide Level due to Renal Impairment on DKD/DR

Since C-peptide is mainly metabolized and excreted by the kidney, renal insufficiency in various kidney diseases, including DKD, will lead to increased serum C-peptide levels [48, 49]. Thus, the relationship between increased C-peptide levels and increased incidence of DKD may be bidirectional. The present cross-sectional study cannot draw any causal relationship, and direct evidences are needed. On the other hand, the increased serum C-peptide level due to renal impairment might have an impact on DR. Atilgan et al. reported that obesity (visceral fat) increased the risk of DR (OR: 1.060, 95% CI: 1.004–1.119, *P* = 0.035), but the association became insignificant after adjusting for UACR and eGFR, indicating it may be attributable to coexisting renal burden [50]. However, Chung et al.'s study showed that C-peptide protected DR in T2DM independently of eGFR [24].

In brief, further studies are needed to elucidate the relationship between altered C-peptide levels according to renal function and DKD/DR.

### 5.5. Protective Effect of C2/C0 on DKD and DR

In the present study, C2/C0 protected both DKD and DR, which may be attributed to better *β*-cell function and glycemic control, but they remained consistent after adjusting for HbA1c and DM duration and other factors. C2_C0 (calculated as postprandial C-peptide minus fasting C-peptide), which may be less accurate to reflect islet function compared with C2/C0, presented a protective effect for DR, but not for DKD. Our results are consistent with previous studies that reported that a lower *Δ*C-peptide was associated with an increased prevalence and severity of DR [24, 51], but inconsistently, Kim et al.'s study showed that *Δ*C-peptide was also associated with DKD [51]. However, Huang et al.'s study showed that increasing C2/C0 was not associated with the prevalence of DKD or DR [52]. Differences in study participants, sampling, C-peptide measurements, study design, and so on may explain the discrepancy in the findings.

Moreover, no gender difference was found in the present study, consistent with some studies [1, 53]. However, females have a higher risk of centripetal obesity [54], and some other studies reported that the association between DKD or DR and obesity was more significant in female patients than in male patients [8]. Thus, prospective studies are needed to further elucidate the complex associations of obesity with DKD or DR.

## 6. Limitations

We first comprehensively compared the associations between different obesity indices and DKD/DR, and whether they were attributable to C-peptide. In multiple subgroup analysis and sensitivity analysis, the results were robust. However, some limitations in this study must be addressed. First, the participants we included were hospitalized patients, and there was a certain selection bias. Patients in the present study had less severe conditions. Second, this was a retrospective cross-sectional study, and a causal relationship between obesity and DKD or DR could not be established. Third, visceral adiposity was measured through the bioimpedance method, which lacks precision since it only measures a small area of peritoneal fat and is sensitive to abdominal wall tension and respiratory status. Forth, the diagnosis of DKD was not based on renal biopsies since it is invasive and cannot be routinely used. Fifth, we were unable to obtain enough data to grade the severity of DR and thus were unable to analyze the association of obesity with DR severity. Sixth, there may be other potential confounding factors that we failed to adjust, such as the use of hypoglycemic drugs.

## 7. Conclusion

Obesity was a risk factor for DKD, and the effect might be attributable to FCP, which represents insulin resistance. The protective effect of obesity or C-peptide on DR was not independent and might be confounded by multiple factors. Higher C2/C0 was associated with a lower risk of both DKD and DR. Further prospective studies in diverse populations are needed to explore the association and causal relationship between obesity and DKD/DR.

## Figures and Tables

**Table 1:** The characteristics of participants stratified by diabetic complications (DKD or DR).

Variables	DKD+ (*n* = 368)	DKD- (*n* = 774)	*P*	DR+ (*n* = 340)	DR- (*n* = 766)	*P*	Total (*n* = 1142)
Age (years)	58 (51, 66)	55 (48, 62)	<0.05	56.5 (50, 63)	56 (48, 63)	0.186	56 (49, 63)
BMI (kg/m^2^)	26.2 (23.8, 28.2)	25.2 (23.3, 27.7)	<0.05	25.2 (23, 27.7)	25.7 (23.6, 27.9)	0.059	25.6 (23.4, 27.9)
Gender (male%)	57.6	58.4	0.801	57.6	59.3	0.613	58.1
Hypertension history (%)	64.1	39.0	<0.05	52.6	43.7	<0.05	47.1
Smoking history (%)	39.1	38.1	0.734	39.1	39.0	0.959	38.4
Education (≥ high school)	56.9	58.9	0.544	50.0	62.7	<0.05	58.3
DM-duration (month)	99 (36, 180)	54 (6, 123)	<0.05	105 (52, 170)	48 (6, 119)	<0.05	66 (12, 139)
CVD history (%)	23.6	14.0	<0.05	16.8	16.7	0.982	17.1
Insulin ever used (%)	47.1	30.0	<0.05	49.7	28.9	<0.05	35.5
SBP (mmHg)	135 (121, 148)	127 (116, 139)	<0.05	132 (118, 147)	129 (117, 141)	0.014	129 (117, 143)
DBP (mmHg)	76 (66, 84)	74 (67, 81)	0.077	74 (66, 82)	74 (68, 82)	0.571	74 (67, 82)
FBG (mmol/L)	7.16 (5.85, 9.48)	7.08 (5.71, 8.88)	0.222	7.17 (6.00, 9.52)	6.99 (5.62, 8.84)	<0.05	7.1 (5.75, 9.05)
HbA1c (%)	9.1 (7.5, 11.0)	8.9 (7.5, 10.7)	0.330	9.2 (7.7, 11.1)	8.8 (7.3, 10.7)	<0.05	9 (7.5, 10.8)
TG (mmol/L)	1.44 (0.97, 2.15)	1.20 (0.86, 1.85)	<0.05	1.26 (0.86, 1.79)	1.29 (0.91, 2.01)	0.083	1.28 (0.89, 1.95)
TC (mmol/L)	4.54 (3.89, 5.43)	4.59 (3.96, 5.31)	0.768	4.49 (3.84, 5.29)	4.61 (3.96, 5.34)	0.130	4.58 (3.95, 5.33)
HDL (mmol/L)	1.08 (0.93, 1.31)	1.11 (0.94, 1.32)	0.147	1.11 (0.95, 1.33)	1.09 (0.93, 1.30)	0.095	1.10 (0.94, 1.31)
LDL (mmol/L)	2.72 (2.12, 3.34)	2.74 (2.18, 3.36)	0.681	2.63 (2.08, 3.21)	2.79 (2.18, 3.41)	<0.05	2.73 (2.16, 3.35)
UACR (mg/g)	132.5 (61.5, 277.0)	16.5 (8.0, 28.0)	<0.05	40.0 (15.0, 125.5)	23.0 (9.0, 62.0)	<0.05	29.0 (11.0,79.0)
eGFR (ml/min/1.73m^2^)	98.1 (68.0, 118.9)	111.4 (96.1, 128.4)	<0.05	106.2 (84.7, 124.2)	109.8 (93.3, 126.8)	<0.05	108.5 (89.9, 125.9)
WC (cm)	91.5 ± 10.8	88.5 ± 10.2	<0.05	88.5 ± 10.8	89.9 ± 10.3	<0.05	89.5 ± 10.5
HC (cm)	96 (90, 101)	95 (90, 99)	<0.05	94 (89, 99)	95 (90, 100)	<0.05	95 (90, 100)
WHR	0.96 (0.91, 1.00)	0.93 (0.89, 0.98)	<0.05	0.94 (0.89, 0.99)	0.94 (0.90, 0.99)	0.83	0.94 (0.90, 0.99)
VFA (cm^2^)	105 (79, 135)	96 (73, 122)	<0.05	93 (68, 124)	100 (77, 128)	<0.05	98 (75, 127)
SFA (cm^2^)	175 (143, 219)	172 (136, 212)	0.102	169 (131, 207)	176 (144, 218)	<0.05	173 (138, 214)
FCP (ng/mL)	1.29 (0.74, 1.99)	1.03 (0.64, 1.59)	<0.05	1.00 (0.59, 1.56)	1.16 (0.7, 1.81)	<0.05	1.12 (0.66, 1.75)
C2/C0	2.5 (1.7, 3.7)	2.8 (2.0, 4.1)	<0.05	2.3 (1.6, 3.4)	2.9 (2.0, 4.2)	<0.05	2.7 (1.9, 4.0)
HOMA_IR	2.58 (2.12, 3.40)	2.39 (2.02, 3.01)	<0.05	2.40 (2.0, 2.96)	2.50 (2.07, 3.21)	<0.05	2.44 (2.06, 3.17)

Abbreviations: DKD indicates diabetic kidney disease; DR: diabetic retinopathy; BMI: body mass index; DM: diabetes mellitus; CVD: cardiovascular disease; SBP: systolic blood pressure; DBP: diastolic blood pressure; FBG: fasting blood glucose; HbA1c: glycated hemoglobin; TG: triglyceride; TC: total cholesterol; HDL: high-density lipoprotein; LDL: low-density lipoprotein; UACR: urinary albumin to creatine ratio; eGFR: estimated glomerular filtration rate; WC: waist circumference; HC: hip circumference; WHR: waist to hip circumference ratio; VFA: visceral fat area; SFA: subcutaneous fat area; FCP: fasting C-peptide level; C2/C0: the ratio of postprandial C-peptide level to fasting C-peptide level; HOMA-IR: insulin resistance index.

**Table 2:** Associations between obesity indices and the prevalence of DKD or DR in the logistic regression models.

Variables		DKD	*P*	DR	*P*
BMI	Base	1.052 (1.058,1.090)	0.005	0.967 (0.931,1.004)	0.078
	Modify 1	1.050 (1.008,1.094)	0.020	0.986 (0.944,1.029)	0.513
	Modify 2	1.025 (0.981,1.070)	0.275	0.988 (0.945,1.033)	0.587

WHR	Base	25.64 (4.148,158.473)	<0.05	1.080 (0.173,6.735)	0.935
	Modify 1	10.97 (1.250,96.267)	0.031	0.842 (0.096,7.415)	0.877
	Modify 2	3.18 (0.335,30.209)	0.314	0.920 (0.099,8.556)	0.941

VFA	Base	1.006 (1.003,1.009)	<0.05	0.996 (0.993,0.999)	0.023
	Modify 1	1.005 (1.001,1.008)	0.008	0.996 (0.993,1.000)	0.065
	Modify 2	1.002 (0.999,1.006)	0.207	0.996 (0.992,1.000)	0.060

SFA	Base	1.002 (1.000,1.004)	0.078	0.997 (0.995,0.999)	0.007
	Modify 1	1.002 (0.999,1.004)	0.168	0.998 (0.996,1.000)	0.106
	Modify 2	1.000 (0.998,1.002)	0.953	0.998 (0.995,1.000)	0.112

Abbreviations: DKD indicates diabetic kidney disease; DR: diabetic retinopathy; BMI: body mass index; WHR: waist to hip circumference ratio; VFA: visceral fat area; SFA: subcutaneous fat area. Model 1 adjusted for gender, HbA1c, TG, TC, HDL, LDL, smoking history, education, duration of diabetes, and insulin use. Model 2 adjusted for FCP.

## Data Availability

The data that support the findings of this study are available on request from the corresponding author.

## Supplementary Materials

Supplementary Materials Supplementary Table 1 Associations of obesity indexes and DKD stratified by FCP quartiles. Supplementary Table 2 Subgroup analysis of the associations between obesity indices and DKD/DR. Supplementary Table 3 Associations between obesity indices and the prevalence of DKD in the logistic regression models after replacing FCP with the index of insulin resistance (HOMA-IR). Supplementary Table 4 Associations between obesity indices and the prevalence of DKD or DR in the logistic regression models stratified by gender Male (n=664). Supplementary Table 5 Associations between obesity indices and the prevalence of DKD or DR in the logistic regression models after few missing values replaced by the mean (HbA1c, lipids, FCP, subadipose, inneradipose, FBG, weight, height). Supplementary Table 6 Associations between C2_C0 and DKD/DR in the logistic regression models.

## References

[1] Man R. E., Sabanayagam C., Chiang P. P. (2016). Differential association of generalized and abdominal obesity with diabetic retinopathy in Asian patients with type 2 diabetes. *JAMA Ophthalmology*.

[2] Garvey W. T., Garber A. J., Mechanick J. I. (2014). American association of clinical endocrinologists and american college of endocrinology position statement on the 2014 advanced framework for a new diagnosis of obesity as a chronic disease. *Endocrine Practice : Official Journal of the American College of Endocrinology and the American Association of Clinical Endocrinologists*.

[3] Valencia W. M., Florez H. (2017). How to prevent the microvascular complications of type 2 diabetes beyond glucose control. *BMJ (Clinical Research ed)*.

[4] Zhao L., Ren H., Zhang J. (2020). Diabetic retinopathy, classified using the lesion-aware deep learning system, predicts diabetic end-stage renal disease in Chinese patients. *Endocrine Practice : Official Journal of the American College of Endocrinology and the American Association of Clinical Endocrinologists*.

[5] Sabanayagam C., Xu D., Ting D. S. W. (2020). A deep learning algorithm to detect chronic kidney disease from retinal photographs in community-based populations. *The Lancet Digital Health*.

[6] Xu X., Gao B., Ding W. (2021). Retinal image measurements and their association with chronic kidney disease in Chinese patients with type 2 diabetes: the NCD study. *Acta Diabetologica*.

[7] Paterson E. N., Cardwell C., MacGillivray T. J. (2021). Investigation of associations between retinal microvascular parameters and albuminuria in UK biobank: a cross-sectional case-control study. *BMC Nephrology*.

[8] Li W., Gong X., Wang W. (2022). Association of different kinds of obesity with diabetic retinopathy in patients with type 2 diabetes. *BMJ Open*.

[9] Wan H., Wang Y., Xiang Q. (2020). Associations between abdominal obesity indices and diabetic complications: Chinese visceral adiposity index and neck circumference. *Cardiovascular Diabetology*.

[10] Zhu W., Wu Y., Meng Y. F., Xing Q., Tao J. J., Lu J. (2018). Association of obesity and risk of diabetic retinopathy in diabetes patients: a meta-analysis of prospective cohort studies. *Medicine*.

[11] van Leiden H. A., Dekker J. M., Moll A. C. (2002). Blood pressure, lipids, and obesity are associated with retinopathy. *Diabetes Care*.

[12] Lu J., Hou X., Zhang L. (2015). Association between body mass index and diabetic retinopathy in Chinese patients with type 2 diabetes. *Acta Diabetologica*.

[13] Wu Z., Yu S., Kang X. (2022). Association of visceral adiposity index with incident nephropathy and retinopathy: a cohort study in the diabetic population. *Cardiovascular Diabetology*.

[14] Taksali S. E., Caprio S., Dziura J. (2008). High visceral and low abdominal subcutaneous fat stores in the obese adolescent: a determinant of an adverse metabolic phenotype. *Diabetes*.

[15] Gesta S., Tseng Y. H., Kahn C. R. (2007). Developmental origin of fat: tracking obesity to its source. *Cell*.

[16] Shen W., Wang Z., Punyanita M. (2003). Adipose tissue quantification by imaging methods: a proposed classification. *Obesity Research*.

[17] Després J. P. (2012). Body fat distribution and risk of cardiovascular disease. *Circulation*.

[18] Novac C., Radulian G., Orzan A., Balgradean M. (2019). Short update on C-peptide and its clinical value. *Maedica*.

[19] Forst T., Kunt T. (2004). Effects of C-peptide on microvascular blood flow and blood hemorheology. *Experimental Diabesity Research*.

[20] Li X., Zhou Z. G., Qi H. Y., Chen X. Y., Huang G. (2004). Replacement of insulin by fasting C-peptide in modified homeostasis model assessment to evaluate insulin resistance and islet beta cell function. *Zhong Nan Da Xue Xue Bao Yi Xue Ban = Journal of Central South University Medical Sciences*.

[21] Changjie G., Xusheng Z., Feng H., Shuguang Q., Jianwen L., Junzhou F. (2017). Evaluation of glomerular filtration rate by different equations in Chinese elderly with chronic kidney disease. *International Urology and Nephrology*.

[22] Cosentino F., Grant P. J., Aboyans V. (2020). 2019 ESC guidelines on diabetes, pre-diabetes, and cardiovascular diseases developed in collaboration with the EASD. *European Heart Journal*.

[23] Wilkinson C. P., Ferris F. L., Klein R. E. (2003). Proposed international clinical diabetic retinopathy and diabetic macular edema disease severity scales. *Ophthalmology*.

[24] Chung J. O., Cho D. H., Chung D. J., Chung M. Y. (2015). Relationship between serum C-peptide level and diabetic retinopathy according to estimated glomerular filtration rate in patients with type 2 diabetes. *Journal of Diabetes and its Complications*.

[25] Zheng W. (2011). Factor analysis of diabetic retinopathy in Chinese patients. *Diabetes Research and Clinical Practice*.

[26] Ahlqvist E., Prasad R. B., Groop L. (2020). Subtypes of type 2 diabetes determined from clinical parameters. *Diabetes*.

[27] Janowska J., Chudek J., Olszanecka-Glinianowicz M., Semik-Grabarczyk E., Zahorska-Markiewicz B. (2016). Interdependencies among selected pro-inflammatory markers of endothelial dysfunction, C-peptide, anti-inflammatory Interleukin-10 and glucose metabolism disturbance in obese women. *International Journal of Medical Sciences*.

[28] Wang Y., Wan H., Chen Y. (2020). Association of C-peptide with diabetic vascular complications in type 2 diabetes. *Diabetes & Metabolism*.

[29] Song S. J. (2016). Obesity and diabetic Retinopathy. *JAMA Ophthalmology*.

[30] Sabanayagam C., Sultana R., Banu R. (2022). Association between body mass index and diabetic retinopathy in Asians: the Asian eye epidemiology consortium (AEEC) study. *The British Journal of Ophthalmology*.

[31] Price S. A., Gorelik A., Fourlanos S., Colman P. G., Wentworth J. M. (2014). Obesity is associated with retinopathy and macrovascular disease in type 1 diabetes. *Obesity Research & Clinical Practice*.

[32] Hwang I. C., Bae J. H., Kim J. M. (2019). Relationship between body fat and diabetic retinopathy in patients with type 2 diabetes: a nationwide survey in Korea. *Eye (London, England)*.

[33] Dossarps D., Petit J. M., Guiu B. (2013). Body fat distribution and adipokine secretion are not associated with diabetic retinopathy in patients with type 2 diabetes mellitus. *Ophthalmic Research*.

[34] Yaribeygi H., Maleki M., Sathyapalan T., Sahebkar A. (2019). The effect of C-peptide on diabetic nephropathy: a review of molecular mechanisms. *Life Sciences*.

[35] Brunskill N. J. (2017). C-peptide and diabetic kidney disease. *Journal of Internal Medicine*.

[36] Vague P., Coste T. C., Jannot M. F., Raccah D., Tsimaratos M. (2004). C-peptide, Na+,K(+)-ATPase, and diabetes. *Experimental Diabesity Research*.

[37] Landreh M., Johansson J., Jörnvall H. (2013). C-peptide: a molecule balancing insulin states in secretion and diabetes-associated depository conditions. *Hormone and Metabolic Research = Hormon- und Stoffwechselforschung = Hormones et Metabolisme*.

[38] Ido Y., Vindigni A., Chang K. (1997). Prevention of vascular and neural dysfunction in diabetic rats by C-peptide. *Science (New York, N.Y.)*.

[39] Leighton E., Sainsbury C. A., Jones G. C. (2017). A practical review of C-peptide testing in diabetes. *Diabetes Therapy : Research, Treatment and Education of Diabetes and Related Disorders*.

[40] Klein R., Klein B. E., Moss S. E. (1995). The Wisconsin epidemiologic study of diabetic retinopathy. XVI. The relationship of C-peptide to the incidence and progression of diabetic retinopathy. *Diabetes*.

[41] Artunc F., Schleicher E., Weigert C., Fritsche A., Stefan N., Häring H. U. (2016). The impact of insulin resistance on the kidney and vasculature. *Nature Reviews Nephrology*.

[42] Jawa A., Kcomt J., Fonseca V. A. (2004). Diabetic nephropathy and retinopathy. *The Medical Clinics of North America*.

[43] Fort P. E., Losiewicz M. K., Reiter C. E. (2011). Differential roles of hyperglycemia and hypoinsulinemia in diabetes induced retinal cell death: evidence for retinal insulin resistance. *PLoS One*.

[44] Stem M. S., Gardner T. W. (2013). Neurodegeneration in the pathogenesis of diabetic retinopathy: molecular mechanisms and therapeutic implications. *Current Medicinal Chemistry*.

[45] Arar N. H., Freedman B. I., Adler S. G. (2008). Heritability of the severity of diabetic retinopathy: the FIND-eye study. *Investigative Ophthalmology & Visual Science*.

[46] Liu H., Ren J. G., Cooper W. L., Hawkins C. E., Cowan M. R., Tong P. Y. (2004). Identification of the antivasopermeability effect of pigment epithelium-derived factor and its active site. *Proceedings of the National Academy of Sciences of the United States of America*.

[47] Akalın N., Köroğlu M., Harmankaya Ö., Akay H., Kumbasar B. (2015). Comparison of insulin resistance in the various stages of chronic kidney disease and inflammation. *Renal Failure*.

[48] Brier M. E., Bays H., Sloan R., Stalker D. J., Welshman I., Aronoff G. R. (1997). Pharmacokinetics of oral glyburide in subjects with non-insulin-dependent diabetes mellitus and renal failure. *American Journal of Kidney Diseases : the Official Journal of the National Kidney Foundation*.

[49] Moh A., Neelam K., Zhang X. (2018). Excess visceral adiposity is associated with diabetic retinopathy in a multiethnic Asian cohort with longstanding type 2 diabetes. *Endocrine Research*.

[50] Ucgul Atilgan C., Atilgan K. G., Kosekahya P. (2021). Retinal microcirculation alterations in microalbuminuric diabetic patients with and without retinopathy. *Seminars in Ophthalmology*.

[51] Kim B. Y., Jung C. H., Mok J. O., Kang S. K., Kim C. H. (2012). Association between serum C-peptide levels and chronic microvascular complications in Korean type 2 diabetic patients. *Acta Diabetologica*.

[52] Huang Y., Wang Y., Liu C. (2022). C-peptide, glycaemic control, and diabetic complications in type 2 diabetes mellitus: a real-world study. *Diabetes/Metabolism Research and Reviews*.

[53] Klein R., Klein B. E. (2016). Body fat distribution and diabetic retinopathy in people with type 2 diabetes. *JAMA*.

[54] Raman R., Rani P. K., Gnanamoorthy P., Sudhir R. R., Kumaramanikavel G., Sharma T. (2010). Association of obesity with diabetic retinopathy: Sankara Nethralaya diabetic retinopathy epidemiology and molecular genetics study (SN-DREAMS Report no. 8) (SN-DREAMS Report no. 8). *Acta Diabetologica*.

