# Supplementary material for: Association of Obesity Indices with Diabetic Kidney Disease and Diabetic Retinopathy in Type 2 Diabetes: A Real-World Study
Source: J Diabetes Res. 2023 Apr 15;2023:3819830. doi: 10.1155/2023/3819830 (PMC10122582; doi:10.1155/2023/3819830)
Supplement: Supplementary Materials — Supplementary Table 1 Associations of obesity indexes and DKD stratified by FCP quartiles. Supplementary Table 2 Subgroup analysis of the associations between obesity indices and DKD/DR. Supplementary Table 3 Associations between obesity indices and the prevalence of DKD in the logistic regression models after replacing FCP with the index of insulin resistance (HOMA-IR). Supplementary Table 4 Associations between obesity indices and the prevalence of DKD or DR in the logistic regression models stratified by gender Male (n=664). Supplementary Table 5 Associations between obesity indices and the prevalence of DKD or DR in the logistic regression models after few missing values replaced by the mean (HbA1c, lipids, FCP, subadipose, inneradipose, FBG, weight, height). Supplementary Table 6 Associations between C2_C0 and DKD/DR in the logistic regression models. [file 3819830.f1.zip › Supplementary Table 3.doc]

**Supplementary Table 3** Associations between obesity indices and the prevalence of DKD in the logistic regression models after replacing FCP with the index of insulin resistance (HOMA-IR)

| Variables |  | DKD | *P* |
| --- | --- | --- | --- |
| BMI | Base | 1.052(1.058, 1.090) | 0.005 |
|  | Modify1 | 1.050(1.008, 1.094) | 0.020 |
|  | Modify2 | 1.034(0.990, 1.079) | 0.133 |
| WHR | Base | 25.64(4.148, 158.473) | <0.05 |
|  | Modify1 | 10.97 (1.250, 96.267) | 0.031 |
|  | Modify2 | 3.75(0.397, 35.331) | 0.249 |
| VFA | Base | 1.006(1.003, 1.009) | <0.05 |
|  | Modify1 | 1.005(1.001, 1.008) | 0.008 |
|  | Modify2 | 1.003(0.999, 1.007) | 0.094 |
| SFA | Base | 1.002(1.000, 1.004) | 0.078 |
|  | Modify1 | 1.002(0.999, 1.004) | 0.168 |
|  | Modify2 | 1.000(0.998, 1.003) | 0.706 |

Abbreviations: DKD indicates diabetic kidney disease; DR, diabetic retinopathy; BMI, body mass index; WHR, waist to hip circumference ratio; VFA, visceral fat area; SFA, subcutaneous fat area.

Model 1 adjusted for gender, HbA1c, TG TC，HDL, LDL, smoking history, education, duration of diabetes and insulin use. Model 2 adjusted for HOMA-IR.
